# Development of Capacitive-Type Sensors by Electrochemical Anodization: Humidity and Touch Sensing Applications

**DOI:** 10.3390/s21217317

**Published:** 2021-11-03

**Authors:** Joaquim O. Carneiro, Artur Ribeiro, Filipe Miranda, Iran Rocha Segundo, Salmon Landi, Vasco Teixeira, Manuel F. M. Costa

**Affiliations:** 1 Centre of Physics, University of Minho, Azurém Campus, 4800-058 Guimarães, Portugal; jose.miranda@dte.pt (F.M.); id6055@alunos.uminho.pt (I.R.S.); vasco@fisica.uminho.pt (V.T.); 2 Centre of Biological Engineering, University of Minho, Gualtar Campus, 4710-057 Braga, Portugal; arturibeiro@ceb.uminho.pt; 3 Civil Engineering Department, University of Minho, Azurém Campus, 4800-058 Guimarães, Portugal; 4 Federal Institute Goiano, Rio Verde 75901-970, GO, Brazil; salmon.landi@ifgoiano.edu.br

**Keywords:** capacitive-type sensor, sensitivity, anodization, nanoporous anodic alumina

## Abstract

This work describes the development of a capacitive-type sensor created from nanoporous anodic aluminium oxide (NP-AAO) prepared by the one-step anodization method conducted in potentiostatic mode and performed in a low-cost homemade system. A series of samples were prepared via an anodization campaign carried out on different acid electrolytes, in which the anodization parameters were adjusted to investigate the effect of pore size and porosity on the capacitive sensing performance. Two sensor test cases are investigated. The first case explores the use of highly uniform NP-AAO structures for humidity sensing applications while the second analyses the use of NP-AAO as a capacitive touch sensor for biological applications, namely, to detect the presence of small “objects” such as bacterial colonies of *Escherichia Coli.* A mathematical model based on equivalent electrical circuits was developed to evaluate the effect of humidity condensation (inside the pores) on the sensor capacitance and also to estimate the capacitance change of the sensor due to pore blocking by the presence of a certain number of bacterial microorganisms. Regarding the humidity sensing test cases, it was found that the sensitivity of the sensor fabricated in a phosphoric acid solution reaches up to 39 (pF/RH%), which is almost three times higher than the sensor fabricated in oxalic acid and about eight times higher than the sensor fabricated in sulfuric acid. Its improved sensitivity is explained in terms of the pore size effect on the mean free path and the loss of Brownian energy of the water vapour molecules. Concerning the touch sensing test case, it is demonstrated that the NP-AAO structures can be used as capacitive touch sensors because the magnitude of the capacitance change directly depends on the number of bacteria that cover the nanopores; the fraction of the electrode area activated by bacterial pore blocking is about 4.4% and 30.2% for B1 (*E. Coli* OD_600nm_ = 0.1) and B2 (*E. Coli* OD_600nm_ = 1) sensors, respectively.

## 1. Introduction

**Figure 1:**
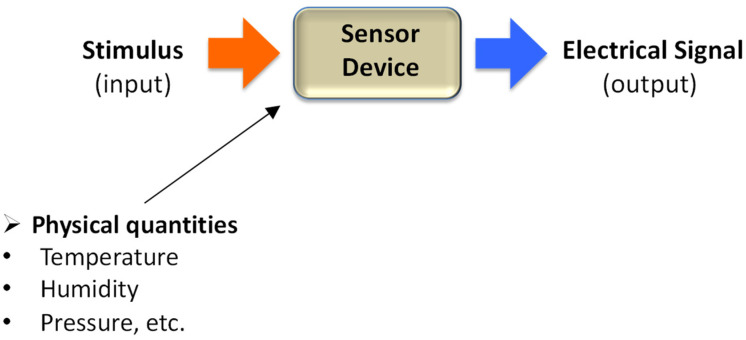
Schematic representation of a sensor’s functioning.

Generally speaking, a sensor device detects and subsequently responds according to some input received from the physical environment. In other words, a sensor is a type of device that converts signals from one source of energy to the electrical domain [1,2,3]. As shown in Figure 1, the input can be any physical property such as temperature, pressure and humidity, among others.

Compared to sensors made from traditional materials, nanomaterials-based sensors bring several benefits in terms of sensitivity and specificity, due to the characteristics of nanomaterials that cannot be found in the bulk material, as they only appear at the nanoscale domain [4]. Due to their small size, the distinctive characteristics and sensitivity improvements of the nanosensors derive from the nanomaterials’ high surface-to-volume ratio and excellent mechanical strength and chemical compatibility [5,6]. In recent years, metal oxide nanoarchitectures have deserved great interest because they have been used in the fabrication of various types of sensors for different applications, since they are mechanically stable, relatively inexpensive and can also work at high temperatures and in severe environments [7,8,9]. Nanoporous anodic aluminium oxide is a morphologically self-organized structure formed by pores with highly ordered hexagonal arrays comprising diameters in the nanoscale domain, and presenting high periodicity and density distribution [10,11]. Among ceramics, it is established that nanoporous anodic alumina is a suitable material to be used in the fabrication of humidity sensors, since its highly uniform nanoporous arrangement greatly increases the surface area available for water adsorption [12,13]; therefore, the performance and sensing capabilities of these sensors depend on their nanoscale morphology. Furthermore, alumina also has excellent properties such as high thermal stability and resistance to chemical attack, hygroscopic features, mechanical strength and availability [14,15,16,17]. Over the last few years, humidity sensors have generated great interest due to the importance of their use in different application areas extending from food quality monitoring, pharmaceutical industry, meteorology, industrial to agricultural fields, in which desirable environmental conditions are monitored by humidity sensors [18,19,20,21]. Moreover, after numerous warnings by food sector regulatory agencies, which report the occurrence of non-compliant foods and cases of food poisoning in people and animals, there is an unquestionable need to monitor bacterial microorganisms such as *Escherichia Coli* (*E. Coli*) and *Salmonella* in agricultural products and prepared/packaged foods using real-time sensing devices such as touch sensors [22,23]. Among the various types of existing sensors (resistive, mass-sensitive, electromagnetic, etc.), capacitive-type sensors are the most popular and of commercial interest because they present a higher sensitivity than the others and have as a competitive advantage their fabrication process, which is simpler [24]. Several techniques exist to produce porous alumina, such as chemical vapour deposition (CVD), sputtering and sol-gel processes [25]. However, there is a common denominator to these production techniques, which is the difficulty in controlling the developed morphology. In addition, the CVD and sputtering methods are technically sophisticated and require the use of very expensive equipment, and the sol-gel method is time-consuming and painful and often the produced samples appear cracked. On the other hand, the electrochemical anodization method not only has the great competitive advantage of being a simple and very low cost fabrication process, but it also enables the development of nanoporous anodic alumina structures, which have potential use in different sensing applications (humidity, bacteria, virus, etc.), where the pore diameter, thickness, porosity and self-aligned cylindrical shape can be controlled in a simple way [26]; for this purpose, it is necessary to properly adjust the anodizing parameters, namely, the type of electrolyte used and its concentration, the applied voltage, the temperature and the anodization time [27,28].

This work describes the fabrication of a dual function capacitive-type sensor (humidity and touch sensing capability) based on nanoporous alumina produced by the one-step anodization method conducted in potentiostatic mode and performed in a custom-made system (this system is a novelty). Unlike other anodization processes, the one-step anodization method has the great advantage of being much less time-consuming and simpler, which for industrial applications is much more advantageous. In this sense, the main objectives of this work can be highlighted as follows: (*i*) provide the design and description of the custom-made anodization system; (*ii*) development of a mathematical model and the electrical equivalent circuits for alumina sensors to estimate humidity condensation inside the pores (humidity sensor) and also to estimate the number of bacteria blocking the pores (touch sensor); (*iii*) evaluate the effect of the used electrolyte on the morphological characteristics of the nanoporous alumina (e.g., pore diameter, interpore distance and porosity); (*iv*) study the effect of different morphological characteristics of nanoporous alumina on capacitive humidity sensing performance; (*v*) study the suitability of nanoporous alumina in the capacitive sensing of bacterial microorganisms, which are potentially associated with the proliferation of diseases.

## 2. Experimental Details

### 2.1. Materials

In this work, all reagents were commercial and were used without further purification.

Acetone, benzine and nitric acid were procured from Quimidroga S.A., Portugal. Phosphoric acid, oxalic acid, sulfuric acid, potassium acetate, potassium carbonate, sodium bromide, magnesium nitrate, cobalt chloride, potassium iodide and sodium nitrate were procured from Merck Life Science S.L.U. (Sigma-Aldrich, Algés, Portugal). Lysogeny broth (LB) liquid medium was procured from Grisp, Portugal.

Aluminium sheets (99.99% purity, 0.25 mm thick) were acquired from Merck (Sigma-Aldrich, Portugal) and used as starting material under which the anodization experiments were carried out. Lead cathodes were procured from a local Portuguese company (Delta Lda.). Gold sputtering target (purity up to 99.99%, 7 cm diameter) was procured form Goodfellow, England.

### 2.2. Fabrication of Nanoporous Anodic Alumina

The initial state of the surface of aluminium substrates plays a major role in the morphology of the anodic layer formed by the anodization technique. Therefore, an effective initial cleaning of the aluminium surface, as well as the use of high purity aluminium as the starting material, should be the essential conditions to ensure the formation of a self-organized assembly of nanopores in the alumina layer. The Al sheets were subjected to a chemical cleaning process. Firstly, the degreasing (i.e., removal of residual mill oil, smut, etc.) of the Al top surface was performed by ultrasonication with acetone/benzine for 10 to 15 min at room temperature (RT). Subsequently, the Al foils were pickled in a 50 g/L of NaOH solution (i.e., an alkaline etch solution) for 1 min at 50 °C to erode the naturally growing aluminium oxide layer (passive layer). Next, the Al test specimens were immersed in a 50% *v*/*v* HNO_3_ solution (i.e., a desmutting agent) for 30 s at 40 °C in order to remove the reaction products formed in the meantime (e.g., complex oxides, hydroxides of aluminium or intermetallic compounds, which are insoluble in the caustic soda etch solution) [29]. The Al sheets were then washed plentifully with distilled water, dried and used as anodes in the electrochemical cell.

**Figure 2:**
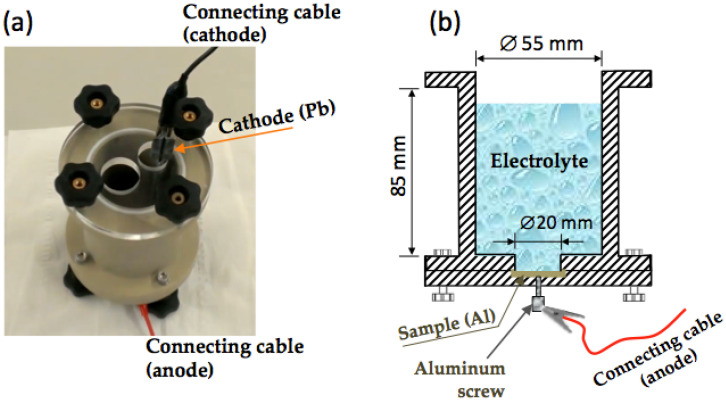
The custom-made electrochemical cell: (**a**) the photograph of the anodization cell showing the position of the cathode and anode and their connecting cables, and (**b**) schematic representation of the anodization cell presenting its main dimensions and showing the circular hole in the bottom base where the aluminium sample (anode) is placed.

The NP-AAO structures were electrochemically produced by using the simple one-step anodization method instead of the conventional two-step anodizing method, which is more complex and consumes more time and chemical reagents. The anodization experiments were implemented in a custom-made anodization cell comprising an anode (pre-cleaned Al sheets) and a cathode (a lead plate), as shown in Figure 2a. The total volume of the anodization cell is about 202 mL, and at its lower base, there is a circular hole with a diameter of 20 mm where the aluminium sample, which acts as the anode, is located. Therefore, during the anodization course, the circular hole is covered by an aluminium sheet, held in place by a tightening screw, to allow the electrolyte solution to bathe its surface (see Figure 2b). On the other hand, the cathode consists of a lead (Pb) plate with a rectangular-type shape and dimensions of 105 × 15 × 4 mm. The volume of electrolyte used in the anodization experiments was 90 mL, which was enough to wet the Pb cathode for a height of about 3/7 of its total length; thus, the wet cathode height/electrolyte volume ratio is around 0.44 mm/mL. Moreover, it should be noted that the volume of 90 mL is, in our case, the optimal electrolyte volume to perform the anodization tests, as it corresponds to the volume that is able to keep the electrolyte temperature stabilized during the anodization time; that is, it corresponds to the threshold volume that minimizes the heat generated (and any local burning problems) from the anodization process.

The morphology of nanoporous alumina depends directly on the anodization parameters, namely the type of used electrolyte and its concentration, the temperature of the electrolytic bath and the applied voltage and anodization time. In this work, three different types of electrolytes were used, namely a sulphuric acid solution (coded as SS), an oxalic acid solution (coded as SO) and a phosphoric acid solution (coded as SP). The different anodization experimental parameters are tabulated in Table 1.

All the Al sheets were only submitted to a one-step anodization by using an EA-PS 3150-04B power supply (from Elektro Automatik). The evolution of the current intensity, I(t), with time was acquired via an EA-UTA 12 analogue interface (from Elektro Automatik) equipped with LabView software.

### 2.3. Design and Fabrication of NP-AAO-Based Capacitive Sensor 

In order to investigate the sensing ability of NP-AAO structures, a parallel-plate capacitor was fabricated where the nanoporous alumina acts as a dielectric-sensing layer. In this design, the non-anodized aluminium sheet at the base works as the bottom electrode of the sensor, whereas a thin gold layer (thickness of 50 nm) directly deposited on the anodized alumina acts as the top electrode. The gold layer was deposited by the sputtering technique using the high-resolution sputter coater Cressington 208HR, equipped with an MTM-20 high-resolution thickness controller. 

**Figure 3:**
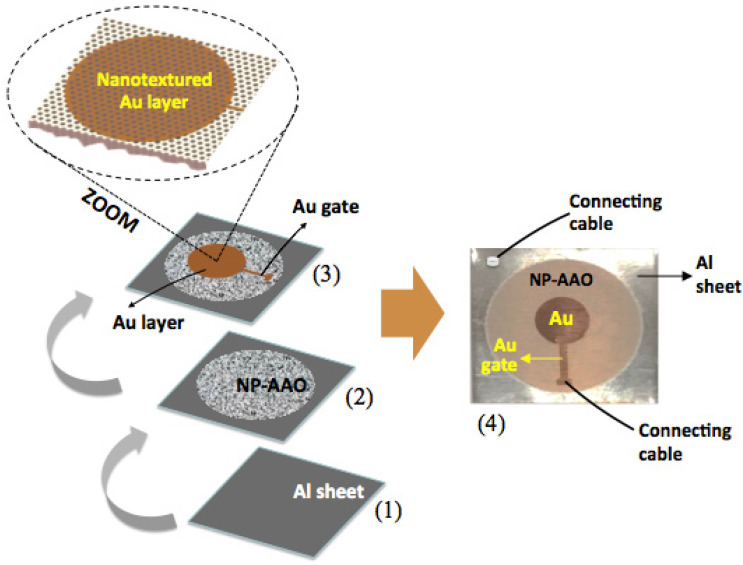
Schematic representation of the capacitive-type sensor: (**1**) the starting Al-sheet, which works as the bottom electrode of the capacitive-type sensor; (**2**) development of the NP-AAO layer by one-step anodization technique; a circular alumina layer (20 mm in diameter) was grown after anodization, comprising the dielectric material; (**3**) a circular nanotextured Au thin layer (diameter of 7.5 mm) is directly deposited by sputtering on the surface of the alumina layer, which acts as the sensor’s top electrode, and (**4**) the photograph of the final architecture of the sensor is presented.

Figure 3 shows the architecture of the capacitive-type sensor, describing its development in sequential steps.

It is emphasized that the direct deposition of a gold thin film on the surface of the nanoporous alumina enables the formation of a nanoporous Au structure with a nanotextured surface topography. This nanoporous and nanotextured Au layer plays an important role, as it has to allow water vapour (application as a capacitive humidity sensor) or a liquid medium of bacterial culture (application as a capacitive touch sensor) to enter through it in order to be adsorbed by the dielectric material, that is, the nanoporous alumina layer. The top Au electrode is of circular geometry with a diameter of 7.5 mm and is linked to a gold compact gate with a length of 5 mm; this arrangement was attained through the use of a specific mask, which was mechanically fabricated from an aluminium foil. The inclusion of a compact gold gate is to prevent the thin nanotextured Au layer from being damaged when applying the electrical connection cable to the sensor’s top electrode. Electrical connections are taken from the two electrodes by using conductive silver glue in the bottom electrode (Al sheet) and a sharp needle positioned in direct contact over the Au–compact gate electrode.

Once the NP-AAO layer acts as an insulator, the design of metal Al, NP-AAO layer and Au nanotextured layer shown in Figure 3 can be exploited to form a metal–insulator–metal (MIM) capacitive-type sensing device (for both humidity sensor and touch sensor applications).

### 2.4. Setup for the Humidity Sensing

A simple, inexpensive and useful method of humidity control is the use of chemical systems, namely, those involving the use of saturated aqueous solutions of simple salts [30,31,32]. Since at a given temperature, particular saturated salt solutions provide a specific relative humidity (RH) value, then the proper selection of a given salt allows obtaining RH values close to the desired ones. In this work, the humidity sensing characteristics of the NP-AAO samples were studied in the range of 23–75% RH (a reasonably wide relative humidity range) using specific saturated salt solutions, according to L. Greenspan’s work [30]. Among all salts presented in the L. Greenspan’s work, we selected those who met the criteria of covering a 23–75% RH range and simultaneously they should be easily accessible at a moderate monetary cost, thus avoiding the use of sophisticated systems or the use of high-cost machines for humidity control.

Table 2 tabulates the saturated salt solutions used in this work and the corresponding RH values at 25 °C [30,31,32]. All salts are anhydrous, except magnesium nitrate and cobalt chloride salts, which are in hexahydrate form.

In order to monitor the sensor’s capacitive behaviour as a function of the RH level, the NP-AAO-based sensor was previously inserted together with the selected saline solution (to generate the desired humidity level) in a sealed desiccator (Duran^®^ vacuum desiccator) with a volume of 0.7 L. 

For the capacitive measurements, each NP-AAO-based sensor was electrically connected to an LCR meter (QuadTech 1920 Precision LCR Meter), passing the connection cables through the upper inlet of the desiccator (taking care to seal this inlet later with a suitable resin). The sensor’s electrical response was acquired with an ac (sinusoidal) input amplitude of 1.0 V (peak to peak) and an applied frequency of 10 kHz; at this frequency, electric dipoles have more time to reorient under the externally applied electric field, thus minimizing the material dielectric losses. It should be noted that each capacitance measurement was only accomplished after waiting an adequate time (about 30 min) to attain the vapour equilibrium condition. The RH level inside the desiccator was recorded by using a commercial and portable RH sensor (Testo 635-2).

### 2.5. Characterization of NP-AAO Morphology

The morphological characteristics of the as-prepared NP-AAO test specimens were analysed by scanning electron microscopy (SEM) through the FEI Quanta 400FEG ESEM/EDAX Genesis X4M microscope. Then, the acquired SEM micrographs were processed by a commercial imaging program (ImageJ) to determine some important morphological parameters of the NP-AAO structures, in particular porosity (*P*), pore diameter (*D*_p_), inter-pore distance (*D*_int_) and pore density (*n*).

### 2.6. Bacterial Culture Medium for Touch Sensing Tests

In this work, the bacterial touch sensing tests were performed only on the SP sample (i.e., anodized in phosphoric acid electrolyte). The obtained morphology for this sample led to the highest average sensitivity in humidity tests, as shown further in Section 3.3. Although we can explore other pore sizes, this work was a proof-of-concept of the touch sensors’ ability to detect microorganisms. In this regard, we used the optimal conditions observed in the humidity tests for the bacterial touch sensing test. In order to investigate the ability of the nanoporous anodic alumina structures to act as a capacitive-type touch sensor, *Escherichia Coli* BL21 (DE3) was used as a model. A bacterial culture was prepared the day before the capacitance experiments by inoculating one colony of *E. Coli* strain BL21 (DE3) into a 100 mL Erlenmeyer flask containing 10 mL of Lysogeny broth (LB). The culture was incubated overnight at 37 °C with constant stirring at 200 rpm. On the day after, the optical density (OD) of the bacterial culture was measured at a wavelength of 600 nm by using a Thermo Scientific GENESYS 20 spectrophotometer. 

Two bacterial suspensions with different OD were prepared (Table 3), to study the effect of the amount of *E. Coli* on the capacitance response of AAO sensors. Since the optical density of the suspensions are directly related to the amount of *E. Coli* present in the bacterial suspension, it is expected a differentiated sensors capacitive response as a function of the number of *E. Coli* present at the surface of the sensor.

To study the bacterial sensing performance, the AAO sensors previously disinfected with increasing concentrations of ethanol were connected to the LCR meter in order to record variations in their capacitive response resulting from changes in the nanotextured surface area of the Au top electrode, due to the presence of *E. Coli*. The sensors’ capacitance was measured for three minutes. After this time, 25 µL of each *E. Coli* suspension (S1 and S2) were added to the surface of the circular electrode of the sensors (B1 and B2). A control was also prepared by adding 25 µL of LB culture medium (S0), without bacteria, to the surface of the sensor (B0). The sensors’ capacitance was recorded each five minutes along thirty minutes of experiment. At the end of this time, an additional 25 µL of bacterial suspension was added to the corresponding sensors (S1→B1 and S2→B2), and the variation in the sensors’ capacitive response was measured.

**Figure 4:**
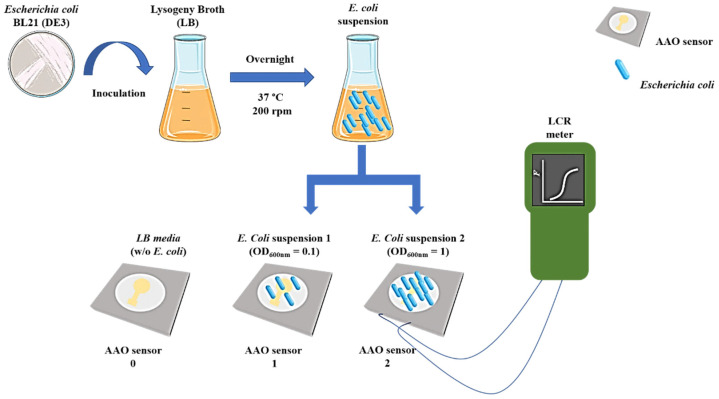
Schematic representation of the experimental sequence for the bacterial touch sensing tests.

The experimental sequence is schematically shown in Figure 4 and the performance of the nanoporous AAO-based capacitive touch sensors was monitored via a capacitance–time plot.

## 3. Results and Discussion

### 3.1. Characteristics of the Anodization Current–Time Curves

**Figure 5:**
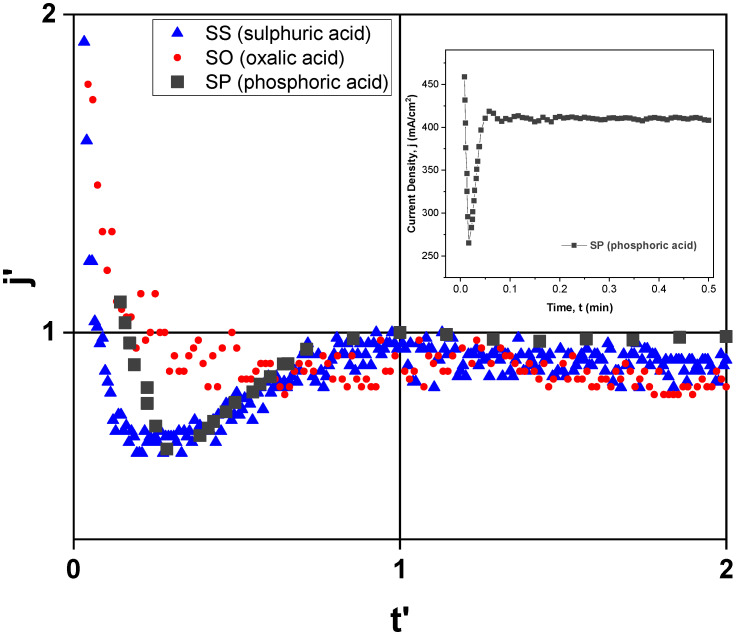
The ordinate and abscissa axes were normalized using the transformations $j^{\prime }=j/j_{\max}$ and $t^{\prime }=t/t_{\max}$, where $j_{\max}$ and $t_{\max}$ are the current density (mA/cm^2^) and time (min) maximum values, respectively. Different values of $\left(t_{\max};j_{\max}\right)$ were found for each electrolyte, namely (1.14 min; 36.5 mA/cm^2^) in sulfuric, (0.37 min; 26.2 mA/cm^2^) in oxalic and (3.6 s; 418.5 mA/cm^2^) in phosphoric acid. The inset shows the curve of current density vs. time of SP sample (anodized in phosphoric acid)—without normalization.

Figure 5 shows the normalized current density curves of the samples SP, SO and SS, anodized in three different electrolytes, namely, sulphuric (21 V), oxalic (40 V) and phosphoric acids (150 V), respectively, which reveals a good similarity with conventional anodization curves conducted in potentiostatic mode, as described by Sulka [33]. The equations describing the formation of alumina porous structures are invariant with respect to the scale transformation $U^{\prime }=kU$ and $t^{\prime }=kt$, as reported by Parkhutik et al. [34]. In this sense, in order to better be able to directly compare the characteristic curves of current density (j) as a function of time (t), a normalization to the curves was performed using $j^{\prime }=j/j_{\max}$ and $t^{\prime }=t/t_{\max}$. For each electrolyte, $j_{\max}$ is the maximum value of current density and $t_{\max}$ is the corresponding time to which the maximum current density occurs, whereas $j^{\prime }$ and $t^{\prime }$ are the dimensionless parameters associated with each of the physical variables. 

Concerning the anodizing process conducted in sulphuric acid, the maximum value of its current density is around 36.5 mA/cm^2^, reached after about 1.14 min (corresponding to about 1.9% of the total anodizing time), while oxalic acid takes around 0.37 min (only about 0.93% of the total anodizing time) to achieve $j_{\max}\approx 26.2\mathrm{mA}/{\mathrm{cm}}^{2}$. On the other hand, the anodization carried out in phosphoric acid (usually known as hard anodization, HA [35]) takes about 3.6 s (now corresponding to 12% of its total anodization time, which is 30 s) to reach $j_{\max}\approx 418.5\mathrm{mA}/{\mathrm{cm}}^{2}$ meaning that, in terms of relative comparison, it takes much longer than those performed in the other two electrolytes. This behaviour was already expected, because as the structure morphology obtained by anodization in phosphoric acid is characterized by large pores (in this work, diameters of 180 nm were found, compared to 40.6 and 18.7 nm obtained in oxalic and sulfuric acids, respectively, and interpore distances of 264.5 nm, compared to 70.6 and 37.1 nm in oxalic and sulfuric acids, respectively), longer times are needed to complete the self-organization of the nanoporous anodic layer. In addition, from Figure 5 it is also possible to make other considerations, namely:

(a) Since oxalic acid is an organic compound, its negative ions $\left(C_{2}O_{4}^{2-}\right)$ are probably barely incorporated into the alumina structure. Compared to the other two electrolytes, a lower concentration of $C_{2}O_{4}^{2-}$ ions in the alumina makes the electric field higher than in the other two electrolytes, thus promoting an earlier nucleation of the pores and consequently making the anodization in oxalic acid a faster process; this is verified in this study, because as mentioned above, the time it takes to reach $j_{\max}$ only corresponds to 0.93% of the total anodization time.

(b) The steady-state current density value $\left(j_{s}\right)$ is different for the three types of produced samples. The sample SP (fabricated in phosphoric acid electrolyte) present the highest steady-state current density average value ($j_{s}^{\left(\mathrm{SP}\right)}=410\mathrm{mA}/{\mathrm{cm}}^{2})$ and the SO sample (obtained from oxalic acid electrolyte) has the smallest, i.e., $j_{s}^{\left(\mathrm{SO}\right)}=21.4\mathrm{mA}/{\mathrm{cm}}^{2}$, while the steady-state current density average value for the SS sample (anodized in sulphuric acid) presents the value of $j_{s}^{\left(\mathrm{SS}\right)}=32.4\mathrm{mA}/{\mathrm{cm}}^{2}$. It is well known that the structure of anodic oxide layer incorporates electrolyte anions (${\mathrm{PO}}_{4}^{3-}$, $C_{2}O_{4}^{2-}$ and ${\mathrm{SO}}_{4}^{2-}$) from the electrolyte used in the anodization [36]. Since $j_{s}$ is directly related to the concentration of incorporated electrolyte anions, the results shown in Figure 5 indicate that the concentration electrolyte anions should higher for the samples that also have a higher steady-state current density value, that is, $j_{s}^{\left(\mathrm{SP}\right)}>j_{s}^{\left(\mathrm{SS}\right)}>j_{s}^{\left(\mathrm{SO}\right)}$. However, the typical content of incorporated anions for some more common electrolytes are of the order of 6–8% in phosphoric acid solution, 10–13% in sulfuric acid and 2–3% in oxalic acid [36].

**Figure 6:**
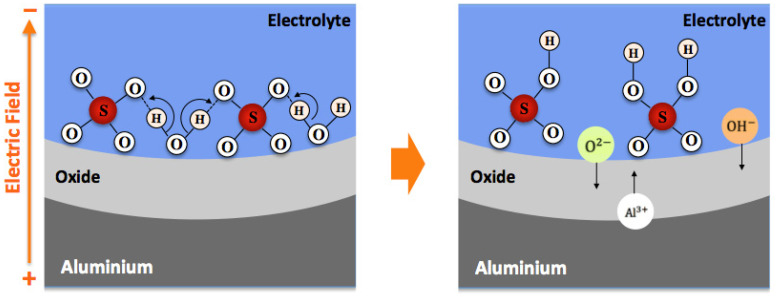
Schematic representation for the generation of $O^{2-}$ and ${\mathrm{OH}}^{-}$ ions at the electrolyte/oxide interface resulting from the interaction of water with adsorbed electrolyte anions (e.g., ${\mathrm{SO}}_{4}^{2-}$ anions).

For the steady-state growth of porous alumina, the role played by the interaction of water with electrolyte anions is shown schematically in Figure 6. It is believed that during anodization, ${\mathrm{OH}}^{-}$ ions are generated in the electrolyte by simple splitting of water, whereas the $O^{2-}$ ions can be formed at the electrolyte/oxide interface from the water interaction with the adsorbed electrolyte anions [37].

For the steady-state growth of the anodic layer, the opposite migration of $O^{2-}/{\mathrm{OH}}^{-}$ and ${\mathrm{Al}}^{3+}$ ions is responsible for the oxide formation, which occurs simultaneously at the electrolyte/oxide and oxide/metal interfaces. At the oxide/electrolyte interface, the presence of electrolyte anions as well as $O^{2-}/{\mathrm{OH}}^{-}$ ions affects the sensing characteristics of capacitive-type humidity sensors because they influence the mechanism of water vapour adsorption on the porous oxide layer; therefore, it is expected that a higher concentration of electrolyte anions should lead to a greater change in capacitance values. This is shown and explained later in Section 3.3.

### 3.2. Analysis of NP-AAO Surface Morphology

**Figure 7:**
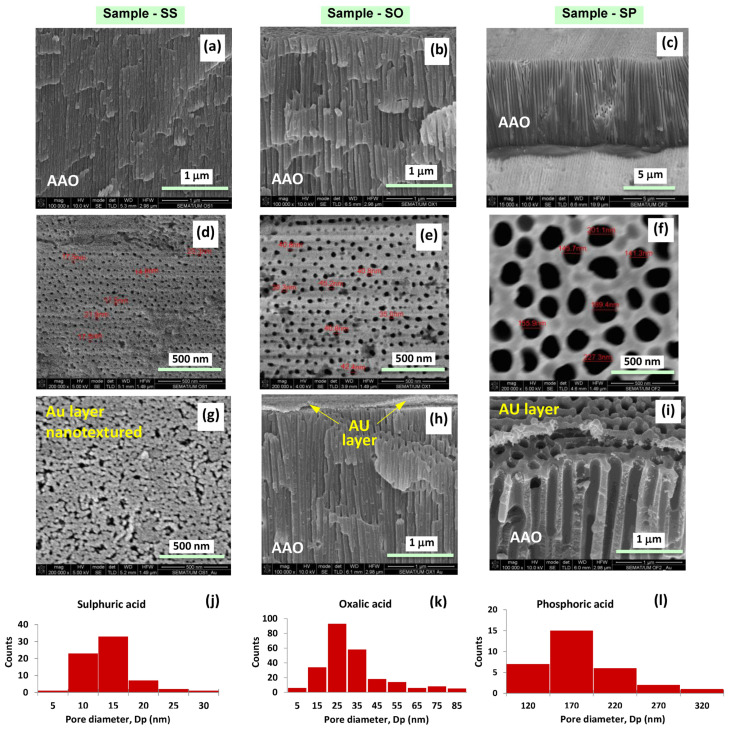
SEM micrographs of samples SS, SO and SP anodized in sulphuric, oxalic and phosphoric acid solutions, respectively; (**a**–**c**) are the cross-sectional SEM micrographs of as-prepared samples SS, SO and SP, respectively while (**d**), (**e**,**f**) are the corresponding surface SEM micrographs; (**g**–**i**) are the SEM micrographs of SS, SO and SP gold-coated samples, respectively and (**j**–**l**) are the size (pore diameter) distribution histogram of samples SS, SO and SP, respectively.

Figure 7 shows the SEM micrographs of bare and gold-coated NP-AAO test specimens. The cross-sectional SEM micrographs of anodized samples in sulphuric, oxalic and phosphoric acid solutions are shown in Figure 7a–c, respectively, while Figure 7d–f displays the corresponding surface SEM micrographs, which also include pore diameter information. Additionally, Figure 7g–i presents the SEM micrographs of gold-coated samples, previously anodized in sulphuric, oxalic and phosphoric acid solution, respectively. Estimates of pore size distribution were determined from the SEM micrographs and are shown in Figure 7j–m as a size (pore diameter) distribution histogram of samples SS, SO and SP, respectively.

From Figure 7, it is possible to observe that some structural morphological features (e.g., pore diameter, inter-pore distance, porosity) of the different samples depend on the type of electrolyte used in the anodization process. Nevertheless, the SEM micrographs confirm that all anodized samples present a common denominator, which is their self-aligned cylindrical shape cells (each containing a pore at the centre) with a high aspect ratio (length-to-pore diameter ratio), long-range ordering and the uniformity and size of the pores at the nanometre scale. Even so, and as noted, pore size varies with the type of electrolyte used and increases with increasing applied voltage, e.g., 21 V, 40 V and 150 V for samples SS, SO and SP, respectively. The increase in pore diameter occurs because in the initial phase of pore formation and growth, a higher voltage leads to a strengthening of the electric field and as a result of a stronger electric field (also stronger at the hemispherical barrier oxide layer at the pore base), the dissolution of the oxide layer is increased.

Figure 7i also reveals that the deposited gold layer on the SP sample surface (anodized with a phosphoric acid solution) is of suitable thickness, as it does not cover the pores underneath it, thus allowing the water vapour molecules (application as a humidity sensor) or the molecules from the liquid bacterial culture medium (application as a touch sensor) to infiltrate through them more easily. On the other hand, observing Figure 7g (SS sample, anodized in a sulphuric acid solution), one can infer that the deposition of the gold layer on the surface of the SS sample led to some obstruction of its pores, which can negatively influence the capacitive response of this anodic structure.

For hexagonal cell porous anodic alumina with a pore inside each hexagon, assuming that each pore is a perfect circle, the porosity, *P* can be expressed as follows [33]:

(1) $$P=\frac{pore\;area}{hexagon\;area}=\frac{\pi }{2\sqrt{3}}{\left(\frac{D_{p}}{D_{i}}\right)}^{2}$$

Here, $D_{p}$ and $D_{i}$ are the pore diameter and the interpore distance (cell diameter) of the alumina nanostructure, respectively, as indicated in Figure 7a. For the alumina nanostructure with a hexagonal distribution of cells, the pore density, *n*, is defined as the total number of pores occupying the surface area of 1 cm^2^, expressed as [33]:

(2) $$n=\frac{10^{14}}{A_{hex}}=\frac{2\times 10^{14}}{\sqrt{3}\;D_{i}^{2}}$$

where $A_{hex}$ is the surface area of a single hexagonal cell (in nm^2^) and $D_{i}$ is expressed in nm. Table 4 presents the structural parameters for the as-prepared NP-AAO samples in which the tabulated values result from the analysis of SEM micrographs shown in Figure 7 by using the image-processing program, ImageJ.

From Table 4 it is possible to verify that the values of the structural parameters of the NP-AAO samples contrast significantly because the samples were anodized with different electrolytes and applied voltages. For example, comparing the SS sample (anodized with a H_2_SO_4_ acid solution and an applied voltage of 21 V) with the sample SP (anodized with a H_3_PO_4_ acid solution and an applied voltage of 150 V), it is observed that their structural parameters are quite different, as for the SP sample the values of *D*_p_, *D*_i_ and *P* are about 9.63, 7.13 and 1.83 times higher than those of the SS sample. Therefore, the SS sample presents a much higher pore density (about 49 times higher) than the SP sample. However, the difference in structural parameters is much smaller when comparing these values for the SS and SO samples. For the SO sample (anodized with an oxalic acid solution and an applied voltage of 40 V) their structural parameters, namely, *D*_p_, *D*_i_ and *P*, are about 2.17, 1.90 and 1.30 times higher, respectively, than the ones obtained for the sample SS; thus, the pore density of the SS sample is only 3.65 times greater than that of the SO sample. In this sense, it is expected that variations of the morphological parameters of nanoporous alumina are responsible for the response of a metal–insulator–metal-based sensor device, in capacitive mode.

### 3.3. Structure and Electrical Equivalent Circuit of a Capacitive-Type Sensor

The pore’s morphology of NP-AAO structures have an important contribution on their capacitive response, because the wetting and formation of a physisorbed layer of water on the pore’s wall surfaces causes a variation in the dielectric constant and consequently, a change in the response of capacitive-type sensors (humidity sensors or touch sensors). Since the very high permittivity exhibited by water is due to the polar structure of its H_2_O molecule, the permittivity of porous dielectric materials is greatly increased with the adsorption of water because the air in the pores is gradually replaced by adsorbed water vapour as the humidity level in the environment increases. In the following, a qualitative model is presented to estimate and explain the sensing behaviour of the fabricated sensor devices, operating in capacitive mode.

**Figure 8:**
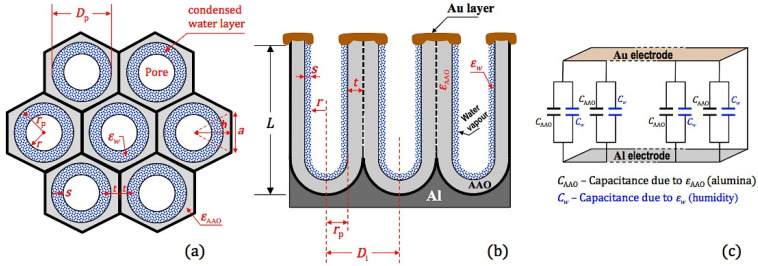
Schematic representation of a nanoporous alumina humidity sensor: (**a**) top view of the NP-AAO array under hexagonal projection geometry; (**b**) cross-sectional (side) view of the NP-AAO based humidity sensor considering individual dielectric constant of alumina and that of the water vapour; (**c**) equivalent circuit of the NP-AAO capacitive-type humidity sensor device.

The nanoporous alumina is a metal–dielectric–metal (MIM) capacitive-type sensing device, as it comprises a cylindrical-shape Al_2_O_3_ porous layer grown on a barrier layer and inserted between two metal electrodes, namely the bottom Al substrate and the top Au thin layer. The top and cross-sectional views of nanoporous alumina humidity sensor are schematically represented in Figure 8a,b, respectively. 

Figure 8a depicts the top view geometrical model of the porous alumina comprising a closed-packed array of hexagonally arranged cells containing pores at each cell centre. Meanwhile, the cross-sectional view of the anodized layer is depicted in Figure 8b, where the cylindrical-shape parallel channels are sandwiched between the top and bottom electrodes. In order to take the effect of the humidity level on the sensor capacitance into account, a qualitative model was developed, in which an annular region (top view) is considered, where each pore is partially filled with a thin layer of water vapour molecules of thickness s. The main parameters of the porous alumina nanostructures are also indicated, namely, the pore radius in the presence of humidity (r), the pore diameter without humidity $\left(D_{p}\right)$, the interpore distance $\left(D_{i}\right)$, the wall thickness (t) and the length of the fine parallel channels (L). In this model, s varies between 0 (no humidity; in this case $r=D_{p}/2$) and $D_{p}/2$ (pore completely filled with water vapour; in this case, r=0). The capacitance resulting only from the contribution of the Al_2_O_3_ anodic layer $\left(C_{\mathrm{AAO}}\right)$, can be expressed as:

(3) $$C_{\mathrm{AAO}}=\frac{\varepsilon_{0}\varepsilon_{\mathrm{AAO}}A_{G}}{L}$$

where $\varepsilon_{0}$ is the dielectric permittivity at 0% RH (i.e., dry environment), $\varepsilon_{\mathrm{AAO}}\;(\sim 9\;\mathrm{at}\;25\;℃)$ [38] and L are the dielectric constant and depth of the NP-AAO layer, respectively, and $A_{G}$ is the area of gold film capped on the surface of the NP-AAO layer, which is related to the porosity of the NP-AAO layer according to the equation:

(4) $$A_{G}=A\left(1-P\right)$$

Here, A $\left(44.2\;{\mathrm{mm}}^{2}\right)$ is the circular area of the gold thin film on flat surface without nanopores and P is the porosity (no humidity) previously defined by Equation (1). Next, one has to consider the effect of the humidity level on the capacitance of the NP-AAO structure. According to Figure 8a, the hexagonal cell geometric parameters h and a can be expressed as (r+s+t) and $\frac{2\sqrt{3}}{3}\left(r+s+t\right)$, respectively. In this sense, an area fraction (f) should be defined, which for a hexagonal cell with an annular region within each hexagon (see Figure 8a) corresponds to the ratio of the annular region area to the area of the hexagon:

(5) $$f=\frac{annular\;region\;area}{hexagon\;area}=\frac{\pi s\left(2r+s\right)}{3ah}=\frac{\pi s\left(2r+s\right)}{2\sqrt{3}{\left(r+s+t\right)}^{2}}$$

Since $\left(r+s+t\right)=\frac{1}{2}D_{i}$ and $\left(2r+s\right)=\left(D_{p}-s\right)$, then the area fraction f can be expressed as:

(6) $$f=\frac{2\pi }{\sqrt{3}}\left[\frac{sD_{p}}{D_{i}^{2}}-{\left(\frac{s}{D_{i}}\right)}^{2}\right]$$

Considering the area of a single hexagon under which a single AAO cylindrical-shape channel resides, and also assuming that the depth of the water vapour layer is around the NP-AAO layer, then the capacitance due to the contribution of humidity $\left(C_{h}\right)$ can be expressed by the equation:

(7) $$C_{h}=\frac{\varepsilon_{0}\varepsilon_{w}\left(fA\right)}{L}$$

where $\varepsilon_{w}$ (~79 at 25 ℃) [39] is the dielectric constant of water.

Figure 8c represents the equivalent electrical circuit of the NP-AAO humidity sensor device. The sensor’s electrical circuit has two components, namely the NP-AAO dry pore wall capacitance $\left(C_{\mathrm{AAO}}\right)$ and an additional capacitor that depends on the surrounding humidity level $\left(C_{h}\right)$. Since both the capacitors (i.e., $C_{\mathrm{AAO}}$ and $C_{h}$) are electrically connected in parallel (see Figure 8c), the effective capacitance $\left(C_{\mathrm{eff}}\right)$ of the NP-AAO sensor device in the presence of humidity can be expressed as:

$$C_{\mathrm{eff}}=C_{\mathrm{eff}}+C_{h}$$

$$\;\;\;\;\;\;\;=\frac{\varepsilon_{0}\varepsilon_{\mathrm{AAO}}A\left(1-P\right)}{L}+\frac{\varepsilon_{0}\varepsilon_{w}\left(fA\right)}{L}$$

(8) $$=\frac{\varepsilon_{0}A}{L}\left[\left(1-P\right)\varepsilon_{\mathrm{AAO}}+f\varepsilon_{w}\right]$$

As an analytical example, it is interesting to analyse two extreme and opposite conditions. In the first condition (dry state), there is no water vapour inside the pores (in this case, s=0, f=0 and $r=D_{p}/2$). In the second condition, the pores are fully filled with condensed water, thus $s=D_{p}/2$, $f=\left(\pi /2\sqrt{3}\right)\cdot {\left(D_{p}/D_{i}\right)}^{2}$ and r=0. Table 5 shows the estimated capacitance values (calculated from Equation (8)) for the two extreme conditions invoked above, which refer to the three different types of NP-AAO fabricated by the one-step anodization technique.

It is interesting to analyse the results shown in Table 5. At the beginning (i.e., the first condition where there is no humidity), it is observed that the capacitance is greater for samples with lower porosity values, i.e., $C_{\mathrm{eff}}\;\left(\mathrm{SS}\right)>C_{\mathrm{eff}}\;\left(\mathrm{SO}\right)>C_{\mathrm{eff}}\;\left(\mathrm{SP}\right)$. This behaviour was already predicted, because in this condition, the effective capacitance only results from the contribution of the AAO dielectric material; therefore, capacitance is evaluated only by applying Equation (3), whose numerator presents higher values for samples with lower porosity. In other words, the area $A_{G}=A\left(1-P\right)$ of the gold film capped on the surface of the NP-AAO layer is higher when the porosity is lower. However, the situation changes when considering the contribution of humidity (second condition).

In the presence of humidity, Equation (8) is applied and as the humidity level increases (increase in thickness s), the annular area $A_{n}=\pi s\left(2r+s\right)$ (see Figure 4) increases (and consequently also increases the area fraction, f), because pores are progressively covered by a layer of water vapour molecules, thus causing a change in the original capacitance. As a comparative analysis, let us first consider the SS sample which is the one with the smallest pores and the lowest porosity value, P. Taking its extreme condition, in which the pores are completely filled with water vapour, the annular area $A_{n}$ becomes equal to the pore circular area $A_{p}=\pi D_{p}^{2}/4$. Therefore, the area fraction f reaches a maximum and equals the original porosity. Under this extreme condition, the capacitance for the SS sample reaches the maximum and even if the humidity level increases, the capacitance does not increase anymore because its pores are already saturated (i.e., fully filled with water vapour). However, for the other two anodized samples (SO and SP) that present a larger pore size and porosity, their capacitance still has room to increase if the humidity level also increases, because their pores are only partially filled with water vapour (i.e., there is still an annular water vapour area).

**Figure 9:**
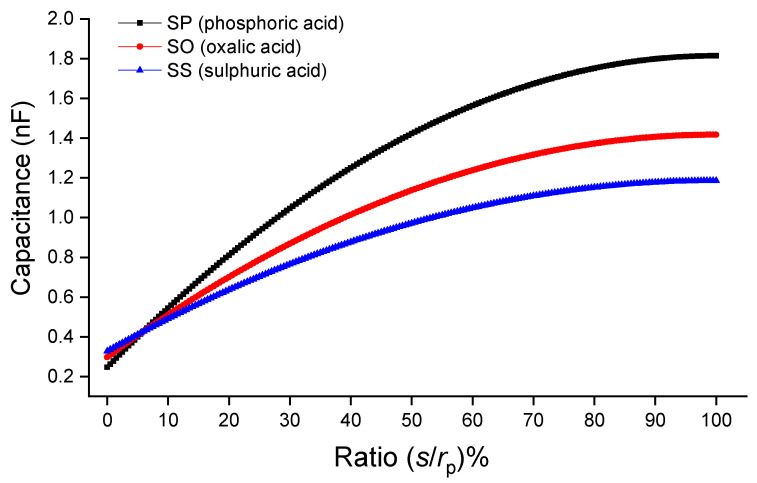
Simulation results for the capacitive response of the sensors with the variation of humidity condensation inside the pores.

Now, let us compare the behaviour of the SS sample with the SP sample, which has a larger pore size and porosity. For the extreme condition of the SS sample, where the pores are completely filled with water vapour (i.e., $s=D_{p}/2=9.35\;\mathrm{nm}$), it is verified that f=P=0.23 and $C_{\mathrm{eff}}=1.186\;\mathrm{nF}$. However, for the same water vapour layer with thickness s=9.35 nm, the SP sample displays an area fraction f=0.083 (well below that of the SS sample), which is still far from its maximum of 0.42 (equal to its porosity value). Therefore, when compared to the SS sample, the capacitance of the SP sample still presents a relatively low value (0.55 nF, as calculated from Equation (8)), as it corresponds to about half of the value displayed by the SS sample. One the other hand, if the humidity level increases again, the area fraction f of the SP sample still has enough room to increase (as well as its capacitance) until it eventually reaches its maximum, which corresponds to a porosity value of 0.42. However, the SS sample can no longer keep up with this increase because its pores are already saturated and therefore its capacitance should remain unchanged even though the humidity level is increasing. In this extreme condition, the capacitance of the SP sample (1.814 nF) already exceeds the value displayed by the SS sample by about 1.5 times, as calculated from Equation (8). Figure 9 compares the capacitive response of the produced samples, as estimated by Equation (8). In order to better perform a direct comparison of the estimated capacitive response for the different samples, the corresponding capacitance values are plotted as a function of the percentage ratio of the adsorbed water layer thickness (s) and the pore radius $\left(r_{p}\right)$.

From Figure 9, it is observed that as the humidity condensation inside the pores increases, that is, for increasing values of the percentage ratio $\left(s/r_{p}\right)\%$, the capacitance values also increase for all samples. Furthermore, it is also perceived that as one progresses towards pore saturation, which occurs for increasing humidity levels (here simulated by high values for s and for the area fraction f), the capacitance values of samples that present a high porosity (yet having low pore density) surpass the capacitance values of the samples that have a low porosity but high pore density. In other words, for the extreme condition (i.e., the second condition indicated in Table 5), there is a change in the order of capacitance values, that is, $C_{\mathrm{eff}}\;\left(\mathrm{SP}\right)>C_{\mathrm{eff}}\;\left(\mathrm{SO}\right)>C_{\mathrm{eff}}\;\left(\mathrm{SS}\right)$. It is clear that the progressive filling (with water vapour) of the area of the originally void regions (air) is the key determining condition for the resulting capacitance of the device.

### 3.4. NP-AAO Humidity Sensor Performance

General speaking, the sensing mechanism underlying humidity sensors is related to the change in capacitance due to the change in the dielectric after adsorption of water vapour.

In most bibliographic references of published works, the sensitivity (S) of a sensor is defined as the slope of its response curve (electrical output signal) as a function of an input stimulus. For a capacitive-type humidity sensor, the slope of the response curve is the ratio of the incremental change in capacitance, ΔC (sensor output signal), to the incremental change in relative humidity, ΔRH (physical property at the input), being expressed by the following equation [40]:

(9) $$S=\frac{C_{y}-C_{23}}{{\mathrm{RH}}_{y}-{\mathrm{RH}}_{23}}$$

where $C_{y}$ denotes the capacitance at the relative humidity of y% RH and $C_{23}$ is the capacitance at the relative humidity of 23% RH, which in this study is also the lower limit of the relative humidity operating range (23≤%RH≤75).

For a more general response, the sensor’s sensitivity can also be expressed in percentage (*S*%), which is given by the equation [41,42]:

(10) $$S\%=\frac{C_{u}-C_{l}}{C_{l}}\times 100$$

Here, the subscripts *u* and *l* represent the values at the upper and lower limits of the operating range, respectively.

In the present work, the sensitivity parameters of the developed sensors were evaluated through Equation (9) because its application provides a more useful understanding of the sensor’s performance, since it carries information about the input and output parameters instead of Equation (10), which only provides output parameters, and therefore, the reader does not have a clear idea about the type of input parameter and its range. It has been reported that the capacitive response arises from the contribution of two main aspects, namely the pore diameter and the presence of anion species, such as $O^{2-}$ and ${\mathrm{HO}}^{-}$, and electrolyte-driven anions, such as ${\mathrm{PO}}_{4}^{3-}$, $C_{2}O_{4}^{2-}$ and ${\mathrm{SO}}_{4}^{2-}$ generated within the pores of the anodic aluminium oxide layer [16]. In an attempt to concisely explain the response of nanoporous alumina exposed to different humidity levels, it was suggested that the electrolyte anions act as a source of high charge density [43], which helps the physisorption of water molecules and leads to the formation of a liquid-like arrangement within the pores, thus changing the dielectric material surrounding the air pore and, consequently, the effective capacitance. 

**Figure 10:**
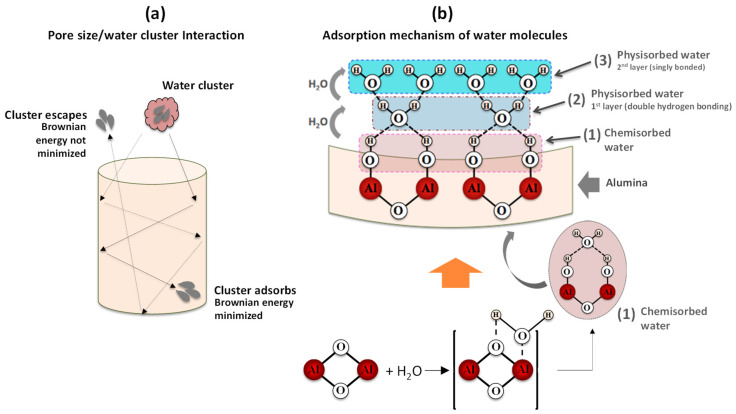
Schematic diagram of interaction of a water molecule with nanoporous alumina surface: (**a**) entry of water molecules through the alumina pores and the subsequent Brownian motion, and (**b**) adsorption mechanism of water molecules with the alumina surface.

Figure 10 is a schematic representation of the effect of pore diameter as well as the mechanism underlying water adsorption by oxide surfaces.

**Figure 11:**
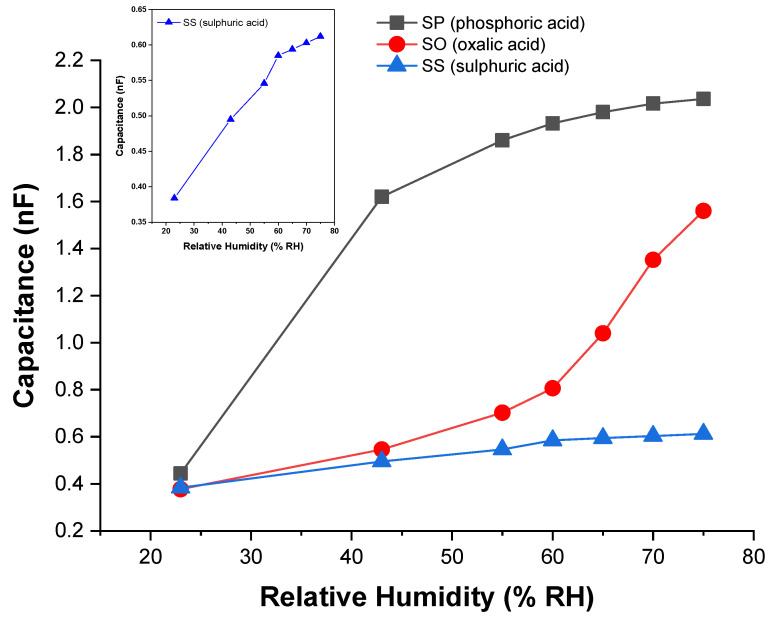
Evolution of capacitance for the sensors SP, SO and SS as a function of relative humidity (% RH).

Regarding the pore size contribution, the process involves three main steps (*i*) the entry of water molecules through the pores; (*ii*) Brownian motion with loss of kinetic energy due to the inelastic collisions of the water molecules with the pore walls or with other molecules, and (*iii*) the adsorption of water molecules on the surface of the pore wall (or eventual exit through the entry door, depending on the molecular Brownian energy). With respect to adsorption, water molecules undergo different chemical and physical processes. If porous alumina is brought into contact with humid air, the water molecules (present at the surface of the base material) are firstly chemisorbed on an activated site of the alumina surface, forming an adsorption complex where the hydroxyl group adsorbs on metal cations present in the oxide surface layer and the proton interacts with an adjacent surface $O^{2-}$ group to form a second ${\mathrm{HO}}^{-}$ group (1) [44]; an increase in humidity level enables another water molecule to be physisorbed (via a double hydrogen bonding of a single water molecule) on the two neighbouring hydroxyl groups, thus forming the first monolayer of physisorbed water (2); as the humidity level continues to rise, the single-layer physisorption changes to multilayer physisorption, where each water molecule will now be singly bonded to a hydroxyl group to form a liquid-like arrangement (3) [44]. This is a necessary condition to enable a change in the capacitive response, since singly bonded water molecules form electrical dipoles that can be freely reoriented under an external applied electrical field, thus resulting in an increase of the dielectric constant. Figure 11 shows the variation of capacitance as a function of relative humidity (%RH) for the different fabricated capacitive-type sensors. The inset included in Figure 11 refers to the capacitive response of the SS sample to facilitate its comparative analysis with the other samples.

From Figure 11, it can be observed that as the humidity level increases, the capacitance value of the sensors also increases, but for the same humidity range (23–75% RH), the change in capacitance for SS, SO and SP samples, is significantly different. The differences in capacitive responses are due to their different pore morphology because it plays an important role in the adsorption/condensation of water vapours in the porous structure of alumina. 

The SP capacitive sensor (with the largest pore diameter) is quite sensitive in the range of 23–55% RH and becomes much less sensitive above 55% RH because its capacitance values increase rapidly with relative humidity up to 55% RH, and afterwards the increase becomes much slower (with a tendency to remain unchanged). This behaviour is directly related to the large pore diameter. We suggest that in the range of 23–75% RH, both small and big water clusters can get easy access into the pores. If only small water clusters existed, their average free path would be high, as they would move in large pores; therefore, they would suffer less inelastic scattering with other clusters, which would result in small losses of Brownian energy and consequently, they would easily escape through the initial entry. However, for this range of humidity values, there should be a strong probability of the presence of a high number of big clusters, so that the mean free path is smaller. Under this condition, the inelastic scattering with other clusters and with the pore walls becomes high, the reduction of Brownian energy is much higher, and the cluster escape is much more difficult. As a result, the cluster adsorption mechanism (in these large-volume pores) is promoted, which causes an increase in the dielectric constant. As the humidity level increases, more and more clusters join to form bigger clusters, more layers of adsorbed water are formed until the saturation condition is reached (when the pores become almost filled), where the dielectric constant no longer changes and therefore, the capacitance also tends to remain unchanged, which seems to happen from 55% RH. 

Concerning the SS sensor, the response characteristic was found to be almost linear, but its capacitance maximum value was remarkably lower than that of the SP sensor (only about 30%) and also very far from the saturation value estimated by Equation (8) (about half). The SS sensor holds the smallest pore diameter, so it acts as a filter that screens for smaller cluster sizes, comparable to its pore diameter. In this sense, irrespective of the humidity level, larger clusters are prevented from entering and do not contribute to changing the dielectric constant. For the low humidity levels (even starting at 23% RH), it can be inferred that fewer clusters are adsorbed because water vapours do not continuously cover the surface. However, as the humidity level increases, more layers of water are successively formed, and the result is a progressive increase in capacitance. We believe that a possible explanation for the low capacitance values recorded for the SS sensor could be related to the inefficient nanotexturing of the gold top electrode; the gold film may have been deposited too thick so that many pores may have been obstructed, as suggested by the SEM micrograph analysis of Figure 7b.

Regarding the SO sensor, and unlike the SP sensor, it is noted that in the range of 23–55% RH the increase in its capacitance is much smaller, while above 55% RH its capacitive response is much more expressive because its capacitance increases significantly (a kind of exponential increase). Compared to the SP and SS sensors, the SO sensor presents an in-between pore diameter; therefore, it is suggested that the SO sensor’s pore morphology is acting as a filter that constrains the entry of the larger molecular clusters into the pores, while smaller ones easily enter and escape through their initial entry without contributing to the adsorption of water vapour molecules. In this sense, it appears that only the statistical population of water clusters that show molecular size compatibility with the pore size of the SO sensor is available to promote a change in dielectric constant upon adsorption, thus leading to a change in its capacitive response, which is much more significant above 55% RH. We assume that above this humidity level, the number of compatible clusters also will increase and become bigger and bulky in weight, thus enabling the coalescence of water vapour molecules on the pore wall surface and subsequent condensation, resulting in a rapid change in capacitive response. Another observation is related to the effect of the incorporation of electrolyte anions in the SO nanoporous oxide structure. Since oxalic acid is an organic acid, it is more than likely that its negative ions are barely incorporated into the alumina layer, because as shown in Figure 5, it exhibits a lower steady-state current density (lower charge density), which does not contribute to potentiate changes in capacitance.

**Figure 12:**
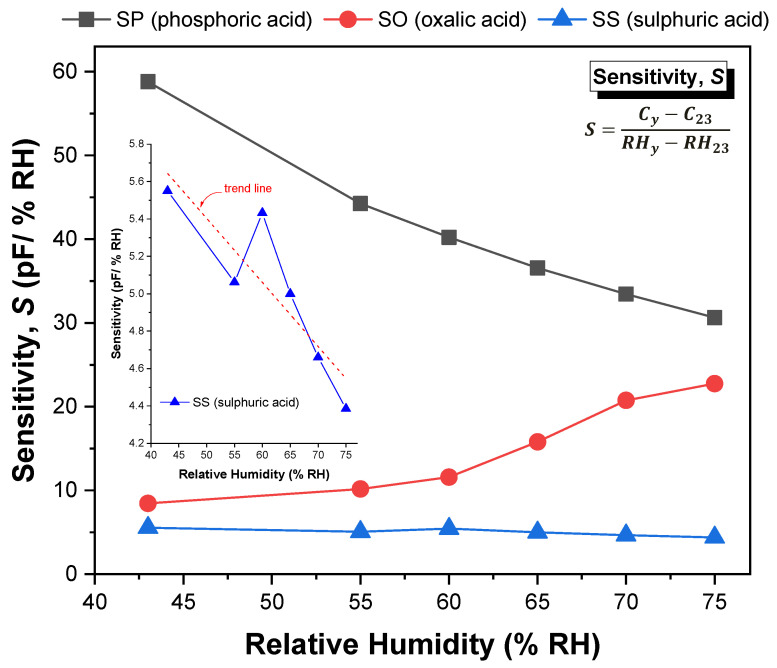
Sensitivity variation of the fabricated sensors as a function of humidity level.

Figure 12 shows the sensitivity values of the SP, SO and SS sensors, determined by Equation (9) in the range of 23–75% RH.

It is understandable that the sensitivity values reflect the behaviour of the capacitive response with the increment in humidity level, as explained in the previous paragraphs. It is observed that for the SP and SS sensors, the sensitivity decreases continuously as the humidity level increases (although SS sensor exhibits a linear-like decrease trend over the entire relative humidity range), while for the SO sensor the sensitivity increases with the increment in the humidity level (a more substantial increase for RH > 60%). The insert included in Figure 12 refers to the sensitivity of the SS sample to facilitate its comparative analysis with the other samples. The average sensitivity value of the SP sensor reaches up to 39 (pF/% RH), while in the case of the SO and SS sensors, the average sensitivity is around 14.5 and 4.8 (pF/% RH), respectively. These values are of the order of magnitude of those reported in [45], except for the value of the SP sample, which is relatively higher (about twice). The reason for this difference may be related with the structural differences or with a higher change of the dielectric constant due to water adsorption.

### 3.5. Bacterial Sensing Performance of NP-AAO Touch Sensors

For absorbance measurements, OD is a logarithmic measurement of the percentage transmission (%T) and it can be represented by the equation,

(11) $$\mathrm{OD}=\log\left(\frac{100}{\%T}\right)\;\;$$

**Figure 13:**
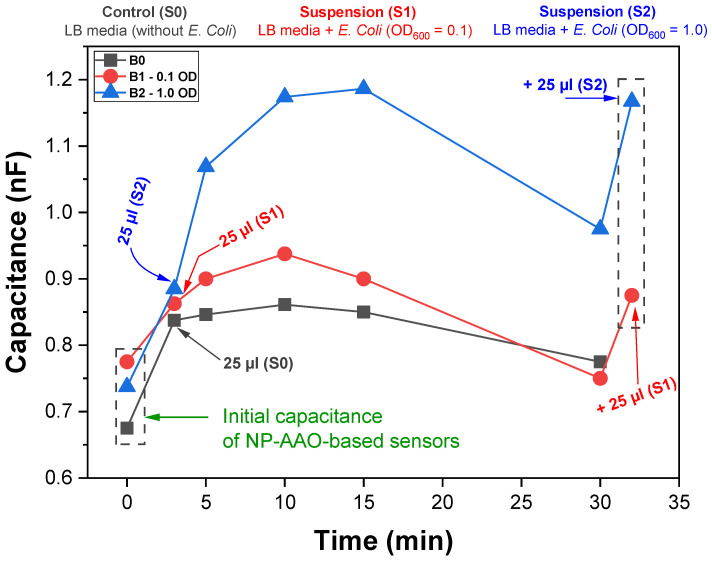
Capacitance variations along time for the NP-AAO touch sensors due to the contact with a suspension of *Escherichia Coli* bacteria.

This means that a sample with 1 OD allows 10% of light to be transmitted through the sample. Figure 13 shows the variation of sensors capacitive response along time for samples B0 (control), B1 (*E. Coli* OD_600nm_ = 0.1) and B2 (*E. Coli* OD_600nm_ = 1.0).

Regardless of the presence/absence of *E. Coli*, an increase of the capacitance was observed after the addition of the suspensions to the surface of the AAO sensors for all the tested conditions. However, this capacitive response was differentiated according to the amount of *E. Coli* present in the bacterial suspension, with the highest capacitance values being recorded for the suspension with the highest amount of *E. Coli* (S2). Although the increase of the capacitive response varied according to the amount of *E. Coli* on the surface of the touch sensors, there was a slow and continuous decrease of the capacitance values after 15 min for all the samples. This phenomenon was most likely related with the evaporation of water from the suspensions. It is noteworthy that the capacitive response was recovered after addition of an equal amount of bacterial suspension to sensors B1 and B2, pointing out the influence of the presence of water on the sensors’ capacitive response (water has a high dielectric constant). For touch sensor applications, it is common to define the touch sensor sensitivity $\left(S_{T}\right)$ as the ratio of the percentage change in capacitance and the area *A* of the gold top electrode (i.e., the gold circular area of the flat surface without nanopores) [23]. Thus, using Equation (10) and dividing it by the area *A* of the top electrode, the sensitivity of the touch sensor is given by the expression:

(12) $$S_{T}=\frac{\Delta C\%}{A}$$

where the percentage change in capacitance is expressed as

(13) $$\Delta C\%=\frac{C-C_{0}}{C_{0}}\times 100$$

Here, C is the capacitance of the touch sensor at a particular time instant and $C_{0}$ is its initial capacitance, i.e., the touch sensor capacitance measured before adding the bacterial suspension. The measurement results presented in Figure 13 show that the B2 touch sensor has a capacitance change of 42.8% (on average), while the B1 touch sensor presents a capacitance change of about 16.1%. This demonstrates that the B2 touch sensor with the addition of the S2 bacterial suspension, is more sensitive $\left(S_{T}=0.97\;\Delta C\%/{\mathrm{mm}}^{2}\right)$ than the B1 sensor $\left(S_{T}=0.36\;\Delta C\%/{\mathrm{mm}}^{2}\right)$. These values are aligned with those reported by Hong et al. [23], who obtained a touch sensor with a sensitivity of about $0.21\;\Delta C\%/{\mathrm{mm}}^{2}$.

The increase in capacitance after the addition of suspensions to the NP-AAO sensors’ surface can be attributed to a change on the effective surface area of the nanotextured top electrode. Actually, considering that bacteria can play the role of an electrically conductive object, it is expected that as they come into contact with the top nanotextured surface of the gold electrode, (air-containing) alumina nanopores will be covered and this causes a change in capacitance. The area change will be AP and therefore the change in capacitance can be determined as:

(14) $$\Delta C_{\mathrm{air}}\;=\frac{\varepsilon_{0}\varepsilon_{\mathrm{air}}\left(AP\right)}{L}$$

**Figure 14:**
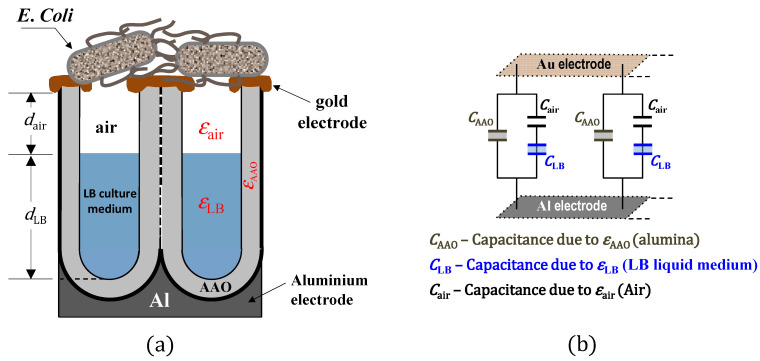
Schematic representation of the NP-AAO capacitive-type touch sensor: (**a**) cross-sectional (side) view of the nanoporous alumina structure where the volume inside each pore is partially filled by the LB culture medium and air, and (**b**) equivalent circuit of the NP-AAO capacitive-type touch sensor device.

However, bacteria (objects) are added to the gold nanotextured surface through a culture medium that essentially consists of a suspension of water containing the nutrients and salts necessary to keep the bacteria alive. In this sense, the increase in capacitance recorded in sensors B1 and B2 is probably associated with the liquid infiltrated into the nanoporous channels of the NP-AAO sensors structure, which should partially replace the initial air. As the exposure time increases, the *E. Coli* bacteria deposit on the nanotextured Au surface and cover some nanopores containing the culture medium. The presence of bacteria on the surface of the gold top electrode, blocking the pores, will contribute to the appearance of an increased number of new capacitors. Therefore, each individual pore should act as two series-connected capacitors, where the total change in effective capacitance has the contribution of the LB culture medium and the air, as shown schematically in Figure 14.

The sensor circuit has three components: the NP-AAO dry pore wall capacitance $\left(C_{\mathrm{AAO}}\right)$ and two additional capacitors (series-connected) causing the change in capacitance due to the presence of two different physical media that are distinguished by different dielectric constants, namely, the dielectric constant of the culture medium $\left(\varepsilon_{\mathrm{LB}}\right)$ and the air $\left(\varepsilon_{\mathrm{air}}\right)$. The effective capacitance $\left(C_{\mathrm{eff}}\right)$ of the NP-AAO touch sensor can be expressed as:

(15) $$C_{\mathrm{eff}}=C_{\mathrm{AAO}}+\Delta C$$

Here, $\Delta C=\left(C_{\mathrm{Air}}\times C_{\mathrm{LB}}\right)/\left(C_{\mathrm{Air}}+C_{\mathrm{LB}}\right)$ where $C_{\mathrm{air}}=\left[\varepsilon_{0}\varepsilon_{\mathrm{air}}\left(AP\right)\right]/d_{\mathrm{air}}$ and $C_{\mathrm{LB}}=\left[\varepsilon_{0}\varepsilon_{\mathrm{LB}}\left(AP\right)\right]/d_{\mathrm{LB}}$. These new capacitors will increase the capacitance performance of the AAO sensors. The differences observed on the capacitive response for sensor B2 and sensor B1 are thus related with the number of *E. Coli* capacitors on the surface of the sensor. The increase in capacitive response of sensor B2 is greater than that recorded for sensor B1, since the number of bacteria present in bacterial suspension 2 (OD_600nm_ = 1) is much higher than that in bacterial suspension 1 (OD_600nm_ = 0.1).

In order to justify this statement and to get an idea about the difference in the number of bacteria present on the surface of each sensor, it is possible to perform a simple exercise where the following conditions are assumed:

(a) For each sensor, every active pore is assumed to be blocked by bacteria and fully filled with LB culture medium (i.e., the pores no longer contain air inside);
(b) For each sensor, the time instant in which the effective maximum capacitance value is registered, that is, 0.938 nF and 1.18 nF for sensors B1 and B2, respectively, is considered.

Under these conditions, the change in capacitance is only due to the infiltration effect of the culture medium into the pores. Therefore, $C_{\mathrm{eff}}=C_{\mathrm{AAO}}+\varepsilon_{0}\varepsilon_{\mathrm{LB}}A^{\prime }/L$, where $A^{\prime }$ is total area of the pores covered by bacteria and fully filled with LB culture medium. In this sense, for each sensor, it is possible to estimate the percentage fraction of the electrode area that is responsible for the increase in the sensor’s capacitance, which is about 4.4% and 30.2% for sensors B1 and B2, respectively, clearly demonstrating that the number of bacteria on the surface of sensor B2 is much higher than on sensor B1.

**Figure 15:**
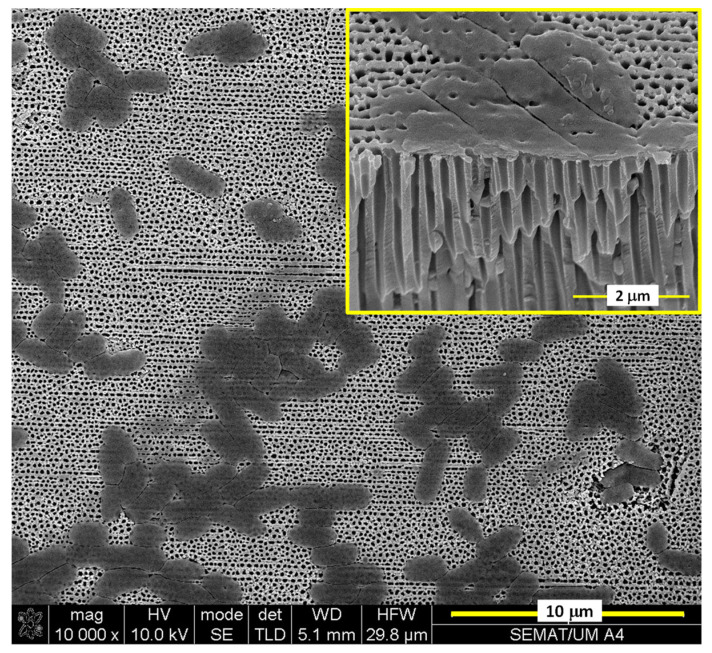
SEM micrograph showing *Escherichia Coli* microorganisms on the surface of the B1 touch sensor’s nanotextured Au top electrode.

Figure 15 is the SEM micrograph of the B1 touch sensor covered by the *Escherichia Coli* population for the exposure time (10 min) at which the maximum capacitance value was recorded.

The SEM micrograph clearly shows that there are large areas on the surface of the B1 touch sensor that are not covered by the bacterial population, which obviously limits the capacitance value measured for this sensor. The inset included in Figure 15 is the cross-sectional view of the SEM micrograph in which the coverage of a certain number of pores by the bacterial population is clearly observed.

## 4. Conclusions

This work reports the successful development of capacitive-type sensors based on nanoporous aluminium oxide (NP-AAO) fabricated through a one-step anodization method by using a homemade anodization cell. The underlying principle of the NP-AAO fabrication and the effect of different anodic layer morphologies (pore size, interpore distance and porosity) on the humidity and touch sensing characteristics were investigated in detail. In the first test case (related to humidity sensing application), nanoporous alumina-based sensors revealed humidity sensing characteristics that depended on pore size. It was found that sensors prepared via anodization in a phosphoric acid solution (i.e., SP samples having the largest pore diameter) were quite sensitive in the range of 23–55% RH, and less sensitive above 55% RH, while the sensor formed in a sulfuric acid solution (i.e., SS samples presenting the smallest pore diameter) showed a linear-like characteristic curve for the capacitance; however, its maximum capacitance value was remarkably lower than that of the SP sensor (it was only about 30%) and also far from the saturation value estimated by the mathematical model developed here and obtained from the electrical equivalent circuit. On the other hand, the sensor formed in oxalic acid solution (i.e., SO samples, with an intermediate pore diameter) was more sensitive in the range above 55% RH. The mechanism responsible for the differences in humidity sensors performance is directly related to the molecular kinetics of water vapour molecules when they interact with anodic structures with different pore diameters; in addition, a complementary/competing mechanism also exists and it is related to the incorporation of electrolyte anions into the alumina layer during the anodization process, which act as a source of high charge density, thus facilitating the physisorption of water molecules; this promotes a change in the dielectric material around the pore and, consequently, the change in effective capacitance.

In the second sensing test case (capacitive touch sensing application), it was demonstrated that sensors prepared via anodization in a phosphoric acid solution were quite sensitive, as they were able to effectively detect the presence of different amounts of *E. Coli* bacteria (simulation model microorganism), where the corresponding mechanism was directly related to the change in capacitance of the sensor. It was found that the sensors capacitance change was 42.8% and 16.1% for the B2 (*E. Coli* OD_600nm_ = 1.0) and B1 (*E. Coli* OD600nm = 0.1) sensors, respectively; this means that the B2 touch sensor is more sensitive $\left(S_{T}=0.97\;\Delta C\%/{\mathrm{mm}}^{2}\right)$ than the B1 sensor $\left(S_{T}=0.36\;\Delta C\%/{\mathrm{mm}}^{2}\right)$. In the future, we aim to explore the loading effect of different microorganisms (bacteria and yeasts) in touch sensors with different pore sizes to evaluate the dependence of sensors’ capacitive response with microorganisms loading and pore size. This will allow the tuning of the sensors according to the microorganism to be detected.

In this work, for biological applications, it was shown that capacitive touch sensors could be useful tools and help detect the presence of pathogenic microorganisms that potentially induce diseases in humans and animals.

## Figures and Tables

**Table 1:** The anodization experimental parameters used for the production of NP-AAO with different morphologies.

Sample Code	Electrolyte Type/Concentration (M)	Electrolyte Temperature (°C)	Applied Voltage (V)	Anodization Time (min)
SS	0.3 M H_2_SO_4_	RT	21	60
SO	0.3 M H_2_C_2_O_4_	RT	40	40
SP	0.3 M H_3_PO_4_	1	150	0.5

**Table 2:** Identification of saturated salt solutions used to generate different values of humidity levels at 25 °C.

Name of Saturated Salt Solution	Relative Humidity, %
Potassium acetate	23
Potassium carbonate	43
Sodium bromide	55
Magnesium nitrate	60
Cobalt chloride	65
Potassium iodide	70
Sodium nitrate	75

**Table 3:** Optical density (OD_600nm_) of *Escherichia* *Coli* BL21 (DE3) suspensions prepared to evaluate the NP-AAO sensors’ capacitance response with the amount of *E. Coli* present in the bacterial suspension.

AAO Code Sensor	*E. Coli* Suspension (S)	OD_600nm_
BO	0	0
B1	1	0.1
B2	2	1.0

**Table 4:** Structural parameters for the as-prepared NP-AAO samples.

Sample Code	Pore Diameter, *D*_p_ (nm)	Interpore Distance, *D*_i_ (nm)	Porosity, *P* (%)	Pore Density, *n* (Pore/cm^2^)
SS	18.7	37.1	23.0	8.4 × 10^10^
SO	40.6	70.6	30.0	2.3 × 10^10^
SP	180.0	264.5	42.0	1.7 × 10^9^

**Table 5:** Capacitance values of NP-AAO samples estimated by the qualitative model (from Equation (8)).

Sample Code	First Condition—Dry (s=0)	$\mathrm{Sec}\mathrm{ond}\;\mathrm{Condition}—\mathrm{With}\;\mathrm{Humidity}\;\left(s=D_{p}/2\right)$
	$\mathrm{Capacitance},\;C_{\mathrm{eff}}\;\left(\mathrm{nF}\right)$	$\mathrm{Capacitance},\;C_{\mathrm{eff}}\;\left(\mathrm{nF}\right)$
SS	0.327	1.186
SO	0.298	1.417
SP	0.247	1.814
